# Mulberry Anthocyanins Ameliorate DSS-Induced Ulcerative Colitis by Improving Intestinal Barrier Function and Modulating Gut Microbiota

**DOI:** 10.3390/antiox11091674

**Published:** 2022-08-27

**Authors:** Jianling Mo, Jingdan Ni, Ming Zhang, Yang Xu, Yuting Li, Naymul Karim, Wei Chen

**Affiliations:** 1 Department of Traditional Chinese Medicine, Sir Run Run Shaw Hospital, School of Medicine, Zhejiang University, Hangzhou 310016, China; 2 Department of Food Science and Nutrition, Zhejiang University, Hangzhou 310058, China

**Keywords:** mulberry anthocyanins, ulcerative colitis, gut microbiota, IBD, intestinal barrier

## Abstract

Mulberry has attracted wide attention due to its substantial nutritional values. This work first studied the protective effect of mulberry anthocyanins (MAS) on dextran sulfate sodium (DSS)-induced colitis. The mice experiment was designed as four groups including normal mice (Control), dextran sodium sulfate (DSS)-fed mice, and DSS plus 100 mg/kg·bw MAS-fed mice (LMAS-DSS) or DSS plus 200 mg/kg·bw MAS-fed mice (HMAS-DSS). Mice were given MAS by gavage for 1 week, and then DSS was added to the drinking water for 7 days. MAS was administered for a total of 17 days. The results showed that oral gavage of MAS reduced the disease activity index (DAI), prevented colon shortening, attenuated colon tissue damage and inflammatory response, suppressed colonic oxidative stress and restored the protein expression of intestinal tight junction (TJ) protein (ZO-1, occludin and claudin-3) in mice with DSS-induced colitis. In addition, analysis of 16S rRNA amplicon sequences showed that MAS reduced the DSS-induced intestinal microbiota dysbiosis, including a reduction in *Escherichia-Shigella*, an increase in *Akkermansia*, *Muribaculaceae* and *Allobaculum*. Collectively, MAS alleviates DSS-induced colitis by maintaining the intestinal barrier, modulating inflammatory cytokines, and improving the microbial community.

## 1. Introduction

Ulcerative colitis (UC) is one of the two principal subtypes of inflammatory bowel disease (IBD), characterized by mucosal inflammation which extends from the rectum to the colon in a continuous manner [1]. Commonly, UC is manifested clinically as diarrhea, abdominal pain, rectal bleeding, and weight loss. In contrast, Crohn’s disease (CD) is another type of IBD and possessed different feathers as patchy lesions, which may be scattered anywhere in the gastrointestinal tract [2]. The incidence of UC is increasing worldwide, which is lower in developing countries. Emerging data, however, show a remarkable growth in the incidence of UC in developing countries [3], although the exact pathogenesis of UC remains unclear, involving immune dysregulation, genetic susceptibility, environmental factors, and the microbiome [4]. Gut microbiome is considered as a modifiable environmental factor that can influence the immune system and seems to be an important target of the inflammatory response [5]. Many factors may affect the gut microbiota, such as mode of delivery at birth, breastfeeding, antibiotic use, infection and diet, which also can promote the development of UC [6].

The intestine is the main habitat of microbiota, which mostly consists of *Bacteroidetes* and *Firmicutes* [7]. Gut microbiota participate in many physiological functions of the host, such as food digestion, nutrient metabolism and energy supply. Accumulated clinical and experimental evidence of IBD have shown the role of intestinal microflora in modulating gut inflammation and barrier function; imbalance of intestinal homeostasis promotes gut inflammation and contributes to a breakdown in intestinal homeostasis [8]. Intestinal permeability may be increased in patients with IBD, which potentially leads to a translocation of intestinal bacteria, eventually exacerbating the inflammatory response and inducing tissue damage [9]. Moreover, gut microbiota metabolites, such as short-chain fatty acids, bile acids, have also shown to be imperative for host prevention and inflammatory regulation [10]. For example, butyrate and niacin have been demonstrated to ameliorate mucosal inflammation in colitis and colorectal cancer [11]. Thus, gut microbiota has become a novel strategy for treating UC. Here, we present a hypothesis that gut microbiota may be a potential target in MAS treating DSS-induced colitis.

Many studies have shown that diet can influence the metabolic health of the host by regulating gut microbiota [12]. Mulberry (*Morus alba* L.) is a moraceous plant, a valuable traditional Chinese medicinal material with a long history of exploitation [13]. A variety of nutrient compounds are found in mulberries such as amino acids, fatty acids and minerals and bioactive compounds [14], including chlorogenic acid, rutin, quercetin and anthocyanin [15]. Moreover, mulberry fruits extracts or their bioactive components, such as anthocyanins, showed multiple bioactive functions in vitro and in vivo, including antioxidant [16], neuroprotective [17], antiatherogenic [18], immune regulatory [19], antitumor, antihyperglycemic, and antihyperlipidemic activities [20]. Modern research has validated many potential health benefits of mulberry anthocyanins MAS, but the therapeutic effects and mechanism of colitis are still unclear.

## 2. Materials and Methods

### 2.1. Materials and Reagents

Dextran sulfate sodium salt (DSS; molecular weight, 36–50 kDa) was purchased from MP Biomedicals Company (Irvine, CA, USA). The antibodies of Muc2, ZO-1, Occludin, and Claudin-3 were used in this study. Other reagents used in this research were of analytical grade.

### 2.2. Preparation of MAS

Fresh mulberry fruits were obtained from a local market in Hangzhou, China. Further, 4 L 70% ethanol aqueous solution containing 0.1% HCL (*v*/*v*) was used to extract 1000× *g* of fresh mulberry. The ethanol extract was then centrifuged at 4000 rpm for 10 min to obtain the supernatant and concentrated at 45 °C. The collected concentrate was sampled on AB-8 macroporous resin column and then eluted using distilled water, 5%, 10% and 20% ethanol aqueous solution (*v*/*v*) orderly. The collected 10% and 20% ethanol eluates were evaporated and then vacuum freeze dried to yield anthocyanin extract powder.

### 2.3. HPLC Analysis of MAS

Then, 10 mg of yield anthocyanin extract powder was dissolved with 1 mL of 0.1% formic acid and centrifuged at 12,000× *g* for 15 min, and 20 μL of supernatant was injected into the HPLC (Dionex Ultimate 3000, Thermo Fisher Scientific, Waltham, MA, USA); the separation was performed on an Ultimate LP-C18 column (4.6 × 250 mm, 5 μm). Elution is performed based on the previous protocol with a little modification [21]. The mobile phase consisted of 0.1% formic acid (solvent A) and acetonitrile (solvent B). The flow rate was 1 mL min^−1^ and the absorbance was detected at 280 nm. The elution protocol was listed as follows: 93–75% A, 0–35 min; 75–35% A, 35–45 min; 35–0% A, 45–46 min; 0% A, 46–50 min; 0–93% A, 50–57 min; 93% A, 57–60 min.

### 2.4. Animal Experiment

All animal experiments were carried out in accordance with China’s guidelines and laws for the use and care of experimental animals (GB/T 35892-2018 and GB/T 35823-2018). Male C57BL/6J mice (20 ± 2 g, 6 to 7 weeks of age) were purchased from Ziyuan Laboratory Animal Technology Co., Ltd. (Hangzhou, China), and housed in specific pathogen-free conditions (temperature, 22 ± 2 °C; relative humidity, 55–60%; and a regular 12/12 h light/dark cycle). All mice were acclimated to the environment for 1 week before the formal experiment, and then randomly divided into four groups (n = 10 for each group). As shown in Figure 2A, the control group (Control) was treated via the administration of distilled water. The model group (DSS) was treated with DSS solution to induce experimental colitis. The first intervention group (LMAS-DSS) was administered MAS via oral gavage at a dose of 100 mg per kg per day for 14 days and treated with DSS solution for 7 days after the administration of MAS for 7 days. Another intervention group (HMAS-DSS) was administered MAS via oral gavage at a dose of 200 mg per kg body weight per day for 14 days and treated with DSS solution for 7 days after the administration of MAS for 7 days. DSS was added to the drinking water from day 7 to 14 of the experimental colitis mice model. Body weights were recorded once daily. Colons were collected at day 17 and stored at −80 °C for further analysis. All analyses were done on the Day 17, which was 3 days post DSS-treatment. All animal experiments were approved by the Committee on Care and Use of Laboratory Animals of Zhejiang University, China (SRRSH202112001).

### 2.5. Pathological Assessment

After the mice were sacrificed, distal colon specimens were fixed in 4% formalin for 24 h and then embedded in paraffin, cut into sections, and stained using hematoxylin and eosin (H&E). After that, the pathological changes in the colon tissue were assessed and scored randomly by an experienced pathologist according to the previous studies [22,23]. Briefly, the total score was calculated from the mucosal thickening score (0–4), the inflammatory cell infiltration score (0–4), the goblet cell depletion score (0–4), the structure destruction score (0–4) and the crypt loss score (0–4). The maximum score was 20.

### 2.6. Evaluation of the Disease Activity Index (DAI) and Colon Length

The DAI was recorded from the eighth day using 3 parameters: body weight loss, stool consistency, and occult blood. The DAI was the mean of the total score of these parameters to evaluate the clinical symptoms of the mice, as previously described [24]. After the mice were euthanized, the colon was excised and washed using PBS solution. The excised colon length was measured and photographed.

### 2.7. Inflammatory Cytokines and Oxidative Stress Markers Analysis

The total protein of colon segments was extracted via homogenizing in 5 volumes (*w*/*v*) of ice-cold physiological saline and then centrifuged at 12,000× *g* for 15 min. The levels of tumor necrosis factor-alpha (TNF-alpha), interleukin (IL)-1β, IL-6 and IL-10 were measured using corresponding ELISA kits. All ELISA kits were purchased from Wuhan Servicebio Biological Engineering Co. Ltd. (Wuhan, China). The levels of oxidative stress markers (superoxidase dismutase (SOD), catalase (CAT), reduced glutathione (GSH), and malondialdehyde (MDA)) were measured using commercial kits (Nanjing Jiancheng Bioengineering Institute, Nanjing, China) according to the manufacturer’s instructions.

### 2.8. Immunohistochemistry (IHC) Analysis

The expression of tight junction (TJ) proteins in the colon were analyzed by IHC [25]. Briefly, the paraffin-embedded sections were deparaffinized and placed in citric acid antigen retrieval buffer with pH = 6.0, then incubated with 3% hydrogen peroxide solution to block endogenous peroxidase. After blocking with 3% BSA, the sections were successively incubated with primary antibodies of ZO-1, occluding, and claudin-3 (1:500, Servicebio, Wuhan, China) and the corresponding enzyme-linked secondary antibodies (Servicebio, Wuhan, China), which were then stained with DAB and hematoxylin (Servicebio, Wuhan, China), and the sections were visualized under an Axio Imager M2 light microscope. In addition, the results were analyzed using the Image J software to count the mean gray value (staining intensity) and the relative quantitative analysis percentage of positive area (staining area) of positive signal.

### 2.9. Immunofluorescence Analysis

Immunofluorescence staining was performed as previously reported with some modification [26]. The paraffin-embedded colon tissue was sliced, deparaffinized and rehydrated, and then the antigen was extracted by microwave in 0.01 M sodium citrate buffer (pH = 6.0), incubated with 2% bovine serum albumin in PBS for 1 h at room temperature to eliminate non-specific binding sites and then incubated with the primary antibody overnight at 4 °C. After that, the sections were washed with PBS and incubated with the corresponding fluorescent conjugated secondary antibody (1:400 diluted in PBS) at room temperature for 2 h, and then stained with DAPI for 10 min. Finally, the sections were photographed under a fluorescence microscope camera system (Nikon Corporation, Tokyo, Japan). The results were analyzed using IPP 6.0 image software for mean fluorescence intensity and semi-quantitative analysis of MUC2 expression.

### 2.10. Fecal DNA Extraction and 16S rDNA Sequencing Analysis

Total stool DNA was extracted and isolated using a DNA Stool Kit (Biomiga, Shanghai, China) according to manufacturer’s protocols. A NanoDrop 2000 was used to measure the purity and concentration of the DNA. The V3–V4 regions of the bacterial 16S rRNA was amplified by the universal primers 338F (5′-ACTCCTACGGGAGGCAGCAG-3′) and 806R (5′-GGACTACHVGGGTWTCTAAT-3′) for 30 cycles with the conditions described previously [27]. The sequencing was performed on an Illumina Novaseq 6000 system according to the standard protocols of Biomarker Technology Co. Ltd. (Beijing, China). The sequencing data were filtered (Trimmomatic v0.33, Chicago, IL, USA), trimmed (cutadapt 1.9.1, MO, USA), and matched (UCHIME v4.2, CA, USA), then used for clustering operational taxonomic units (OTU), diversity analysis, difference analysis, correlation analysis, and function prediction analysis were divided.

### 2.11. Statistical Analysis

Data were expressed as the mean ± standard error of the mean (SEM) after at least 6 independent experiments. A two-way analysis of variance (ANOVA) with Tukey’s multiple comparisons was used to compare pairwise groups in GraphPad Prism 5.0 software (GraphPad Software, San Diego, CA, USA). A statistical difference was considered significant at the values of *p* < 0.05.

## 3. Results

### 3.1. Chemical Properties of MAS

**Figure 1:**
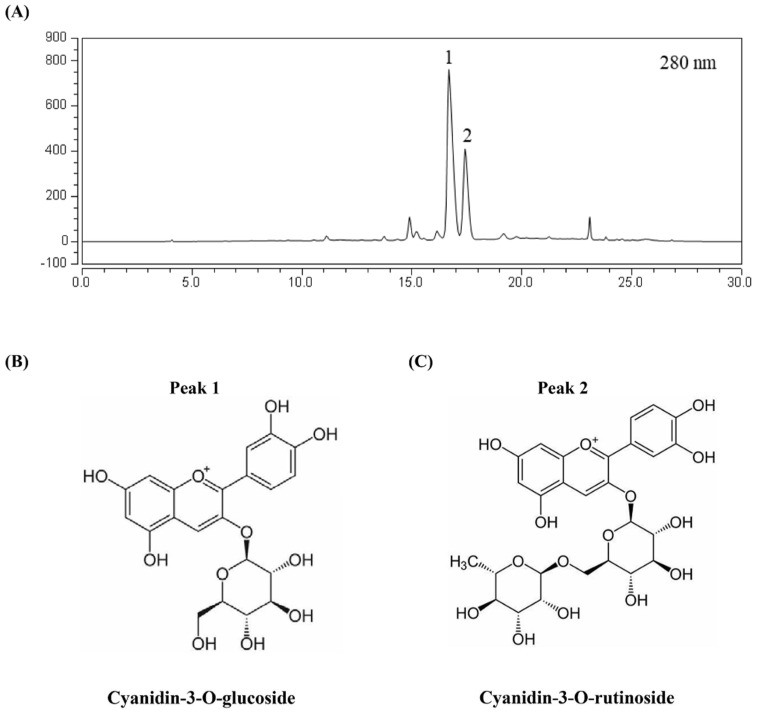
HPLC chromatograms of MAS. (**A**) The absorbance was detected at 280 nm. (**B**) Compound name and structure in peak 1. (**C**) Compound name and structure in peak 2.

MAS were purified according to our previous protocol [28,29]. The main components were shown in Figure 1. Peak 1 and peak 2 were identified as cyanidin-3-O-glucoside and cyanidin-3-O-rutinoside, respectively. Other components in MAS were tentatively identified as quercetin-3-O-rutinoside, quercetin hexoside, kaempferol rhamnosylhexoside and quercetin rhamnosyl hexoside, based on our previous study [30]. Cyanidin-3-O-glucoside and cyanidin-3-O-rutinoside were the main compounds after being purified by macroporous resin, with the contents achieved 74.57% of total mulberry extract calculated by peak area.

### 3.2. MAS Ameliorates Colitis Induced by DSS in Mice

**Figure 2:**
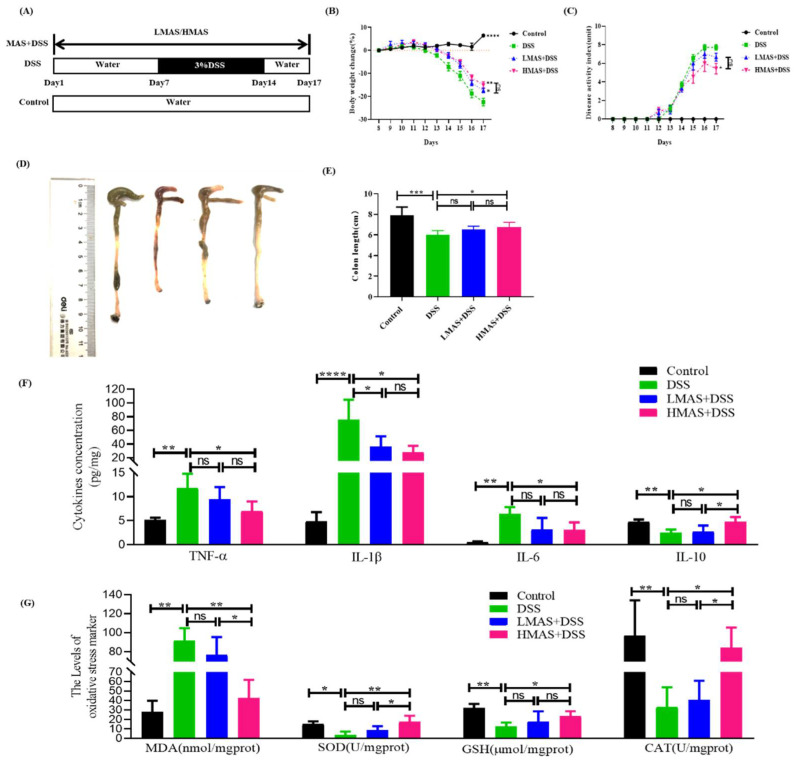
MAS attenuates DSS-induced damage. (**A**) Schematic of experimental design. (**B**) Changes of body weight. (**C**) DAI score. (**D**) Length of experimental mice colon. (**E**) Representative photograph of colons. (**F**) The inflammatory TNF-α, IL-1β, IL-6, and anti-inflammatory cytokine IL-10 levels. (**G**) The levels of oxidative stress markers (MDA, SOD, GSH, and CAT). Here, ns = not significant. Data were expressed as mean ± SD. * *p* < 0.05, ** *p* < 0.01, *** *p* < 0.001, **** *p* < 0.0001, in comparison with the DSS group (n = 10).

Body weight change is an important indicator of disease severity in DSS-induced colitis. The percentage of weight loss from baseline was monitored for 10 days after initiation of DSS treatment. As observed in Figure 2B, the body weight in the control group increased gradually, while the DSS group showed rapid decreases starting at day 4 and sustained until the end of the experiment (*p* < 0.001). However, the decreases were significantly inhibited by treatment with MAS (100 and 200 mg/kg·bw) from day 14 to sacrifice (*p* < 0.05). The body weight of the DSS group significantly decreased, by 22.4%, compared to the control group, but the weight loss in the MAS groups was only 17.3% (LBME+DSS group, *p* < 0.05) and 15.1% (HBME+DSS group, *p* < 0.01). Therefore, MAS possessed a protective role to prevent weight loss.

The DAI score was used to evaluate the progress of DSS-induced colitis. The DAI score for each group was counted as in Figure 2C. The DAI score of the DSS group (7.7 ± 0.28) was increased, significantly higher than the control group (*p* < 0.001), beginning at day 4 of the DSS intervention and continuing until the end of the experiment at day 17, while the score of the HMAS-DSS group (5.7 ± 0.73) was lower than the DSS group (*p* < 0.05). There is no remarkable difference between the LMAS-DSS group and the DSS group (*p* > 0.05). Therefore, MAS has displayed potential protective benefits in treating diarrhea and hematochezia of DSS-induced colitis.

Colon shortening is a feature of colon inflammation in DSS-induced colitis. The DSS group developed severe colon shortening following the administration of DSS for 7 days (*p* < 0.001), while a high dose of MAS (HMAS-DSS group) almost completely maintained the colon length compared to the DSS group (*p* < 0.05) and the effect was not obvious in the LMAS-DSS group (Figure 2D,E). Taken together, MAS possessed protecting potential against DSS-induced colitis.

### 3.3. MAS Decreased Inflammatory Cytokine Production and Suppressed Oxidative Status

Considerable studies have proved that the predominant proinflammatory cytokines, including IL-6, IL-1β, and TNF-α, are the main anti-inflammatory cytokines including IL-10 in patient with UC, which had a deep influence on the development and treatment of UC [31]. The anti-inflammatory effects of MAS against colitis were shown in Figure 2F. According to Figure 2F, the secretion of proinflammatory factors (TNF-α and IL-1β and IL-6) was enhanced dramatically and the secretion of anti-inflammatory cytokines (IL-10) was decreased significantly in the colon of mice after the treatment of DSS compared to the control group (*p* < 0.01). Interestingly, MAS intervention effectively antagonized the changes in cytokines caused by the DSS treatment (*p* < 0.01). Meanwhile, some representative oxidative stress factors were also detected. As displayed in Figure 2G, the SOD, GSH, and CAT were significantly decreased after DSS treatment (*p* < 0.05). However, these parameters were markedly reversed by HMAS treatment (*p* < 0.05) and the levels of MDA were also significantly decreased. Thus, MAS could have the ability to inhibit the inflammatory response and enhance the antioxidant defenses to suppress UC.

### 3.4. MAS Maintains Intestinal Barrier Function and Decreases Gut Permeability

**Figure 3:**
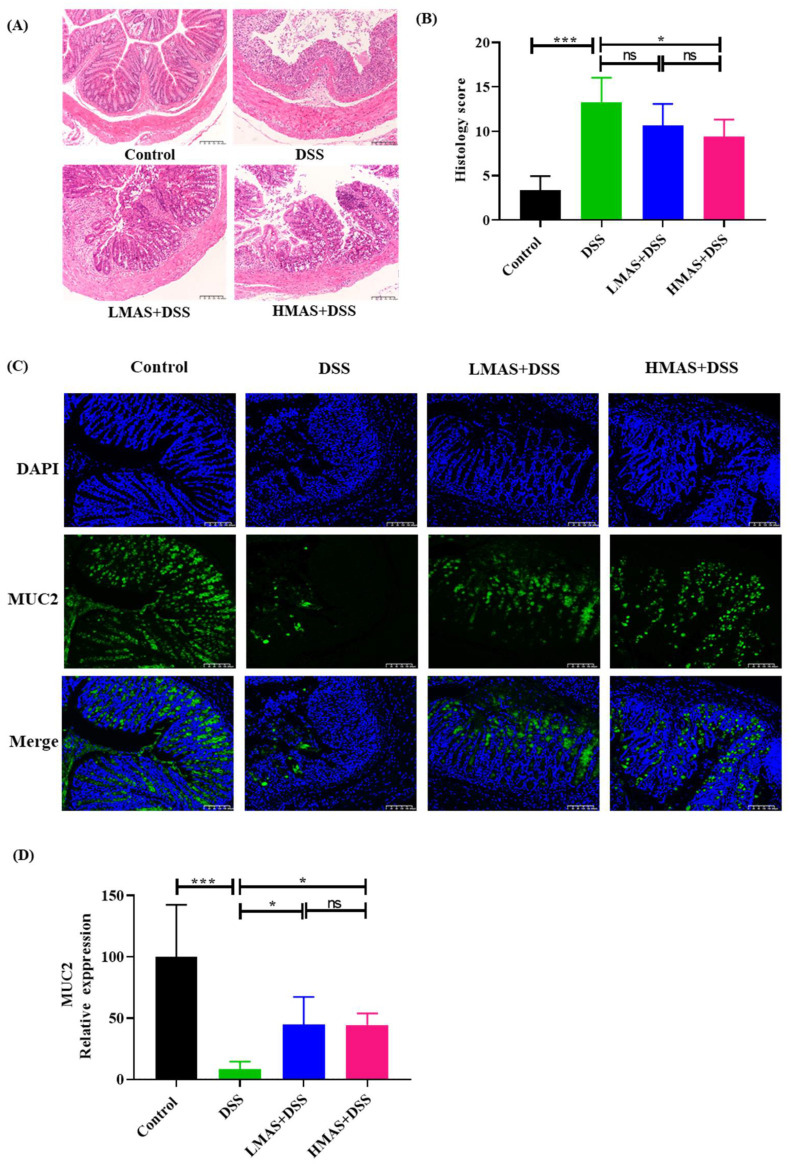
MAS ameliorates intestinal barrier damage in DSS-induced mice. (**A**) H&E stain of colonic tissues sections (200×). (**B**) Pathological scores of colonic tissues sections. (**C**) Muc2 expression in mice colon (200×). The green fluorescence represents the amount of Muc2, and the blue fluorescence is the nucleus stained by DAPI. (**D**) Quantitative analysis of MUC-2. Here, ns = not significant. Data were expressed as mean ± SD. * *p* < 0.05, *** *p* < 0.001, in comparison with the DSS group (n = 10).

Colitis also caused colon pathologic changes. Histological changes were determined by H&E staining. Microscopically, normal histological architecture of the colon was shown in the control group (Figure 3A), and intact epithelium and crypts were observed with neat villi and abundant goblet cells without inflammatory cell infiltration. In contrast, worse erosion, crypt destruction, and submucosal edema were revealed in the colon of the DSS group compared to the control group, which achieved significantly higher disease activity scores (*p* < 0.001). Importantly, as shown in Figure 3B, the HMAS-DSS group exhibited less severe colonic tissue damage, reduced inflammatory infiltration and more intact crypt structures compared with the DSS group (*p* < 0.05); the LMAS-DSS group failed to inhibit histological changes compared to the DSS group (*p* > 0.05).

Mucins, particularly Muc2, are effective barriers for the protection from pathogens and toxins in the intestinal lumen. Muc2 is the predominant mucin gene expressed in colonic goblet cells and is a key factor to maintain colonic health. Here, we determined the protein expression of Muc2 by immunofluorescence. As shown in Figure 3C,D, Muc2 staining (green) for goblet cells was abundant in the colon of the control group, while DSS treatment significantly decreased Muc2 expression (*p* < 0.001), by 10-fold. The treatment of MAS maintained the expression of Muc2 in the DSS-induced colitis, which expressed higher levels of Muc2 than the DSS group (*p* < 0.05). Thus, it shows that MAS protects the integrity of the mucosal layer by inhibiting the down-regulation of Muc2 in the colon of DSS-induced colitis mice.

**Figure 4:**
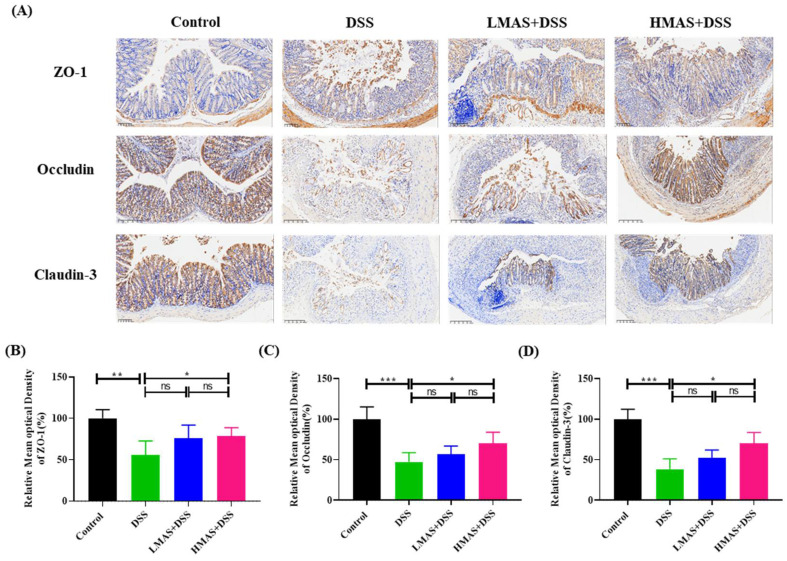
MAS reduced intestinal epithelial cell permeability in DSS-induced mice. (**A**) Immunohistochemistry analysis of ZO-1, Occludin, and Claudin-3; the image magnification is ×200. (**B**–**D**) Immunohistochemical analysis. Here, ns = not significant. Data are shown as the mean ± SD. * *p* < 0.05, ** *p* < 0.01, *** *p* < 0.001, in comparison with the DSS group (n = 10).

Epithelial tight junction (TJ) proteins including ZO-1, occluding, and cladudin-3 are the key markers for epithelial integrity. TJ proteins play an important role in maintaining intestinal barrier integrity and preventing epithelial leakage. Thus, to investigate the effects of MAS on epithelial TJ proteins, we measured the protein level of TJ proteins by IHC. As shown in Figure 4, compared with the control group, the occluding and claudin-3 in the DSS group were almost destroyed and dramatically reduced in the cell membrane and cytoplasm. The ZO-1 protein was discontinuously distributed at the intestinal lumen edge (*p* < 0.001). There were no significant reverse effects for claudin-3, occluding, and ZO-1 expression in the LMAS-DSS group compared to the DSS group (*p* > 0.05), however, a high dose of MAS treatment can hold the expression of TJ protein in DSS-treated mice (*p* < 0.05). Collectively, these results suggest that MAS might affect epithelial integrity by maintaining TJ protein expression in colitis.

### 3.5. MAS Alleviates DSS-Induced Gut Dysbiosis

**Figure 5:**
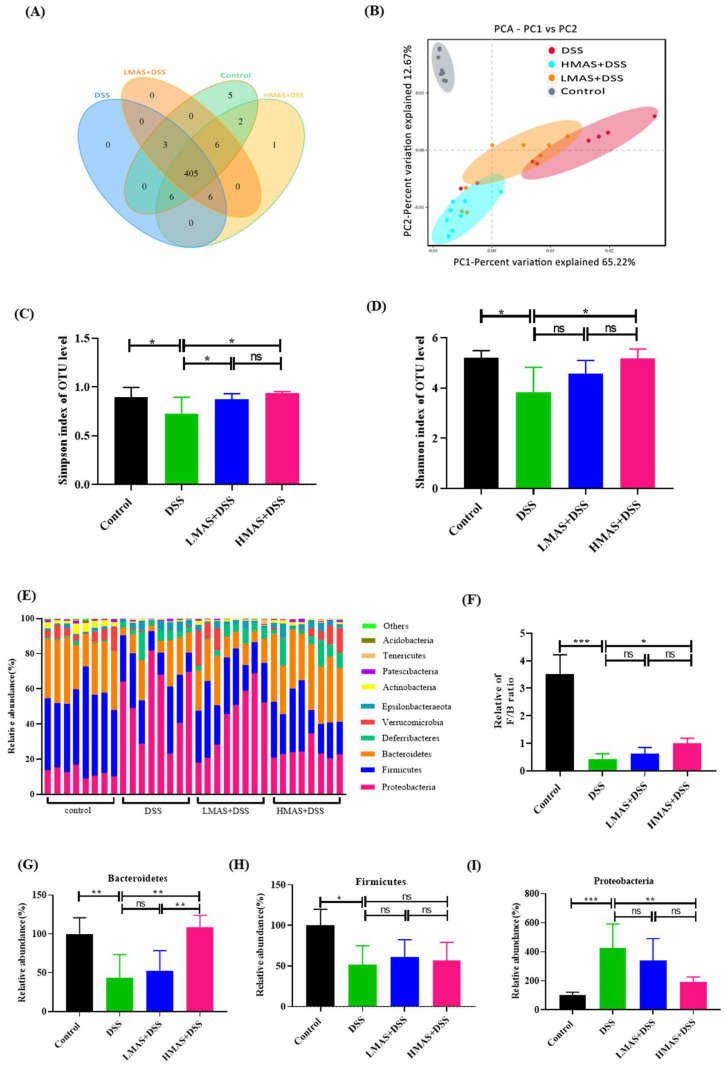
MAS improved the gut microbiota disorder caused by DSS-induced colitis in mice. (**A**) Venn diagram showing the overlap of the operational taxonomic units (OTUs) identified in the gut microbiota among the four groups. (**B**) beta diversity analysis of intestinal microbiota among the four groups using the PCA method. (**C**) Simpson index of all samples. (**D**) Shannon index of all samples. (**E**) Bar chart of the bacterial community composition at the phylum level. (**H**) The *Firmicutes* to *Bacteroidetes* ratio. (**F**,**G**) Relative abundance of *Bacteroidetes*, *Firmicutes* at the phylum level, (**I**) *Proteobacteria* at the phylum level. Here, ns = not significant Data are shown as the mean ± SD. * *p* < 0.05, ** *p* < 0.01, *** *p* < 0.001, in comparison with the DSS group (n = 8).

A Venn diagram was used to identify the common and the characteristic taxa in different groups. As shown in Figure 5A, the numbers of unique operational taxonomic units (OTUs) in the control, DSS, LMAS-DSS, and HMAS-DSS groups were 5, 0, 0, and 1, respectively. All of the groups shared 405 OTUs among gut microbiota. Next, the relative similarity of the gut microbiota composition was visualized using weighted UniFtacPCoA, which showed that the control group, the DSS group and the HMAS-DSS group had different gut microbiota structures, and the LMAS-DSS group and the DSS group had similar gut microbiota structures (Figure 5B), indicating that HMAS-DSS treatment modulated the gut microbiota dysregulation in DSS-induced colitis mice. In addition, compared with the control group, gut microbiota α -diversity was decreased in the DSS group, which was represented by a decrease in Simpson index and Shannon index, while MAS administration inhibited the decrease of the bacterial community richness index (Figure 5C,D).

### 3.6. Key Phylotypes of the Gut Microbiota Altered during MAS Intervention in Colitis Mice

To investigate whether MAS intervention during colitis modulates the composition and structure of gut microbiota, Wilcoxon rank sum test was therefore used to compare the distribution of bacteria at different taxonomic levels among the four groups. The histograms illustrated the species and relative abundance of gut microbiota at the phylum level (Figure 5E). The abundance of *Firmicutes* and *Bacteroidetes* was decreased in the DSS group compared to that in the control group (*p* < 0.05). In contrast, a high dose of MAS treatment holds the abundance of *Bacteroidetes* (Figure 5F,G, *p* < 0.05). This was characterized by the *Firmicutes*/*Bacteroidetes* (F/B) ratio, which is the characteristic of gut microbiota in the DSS group that had a dramatic decrease compared to that in the control group (*p* < 0.001), and this trend was also inhibited by the high dose of MAS treatment (Figure 5H, *p* < 0.05). In addition, the abundance of *Proteobacteria* was also increased due to DSS treatment and was inhibited after the high dose of MAS treatment (Figure 5I, *p* < 0.01).

**Figure 6:**
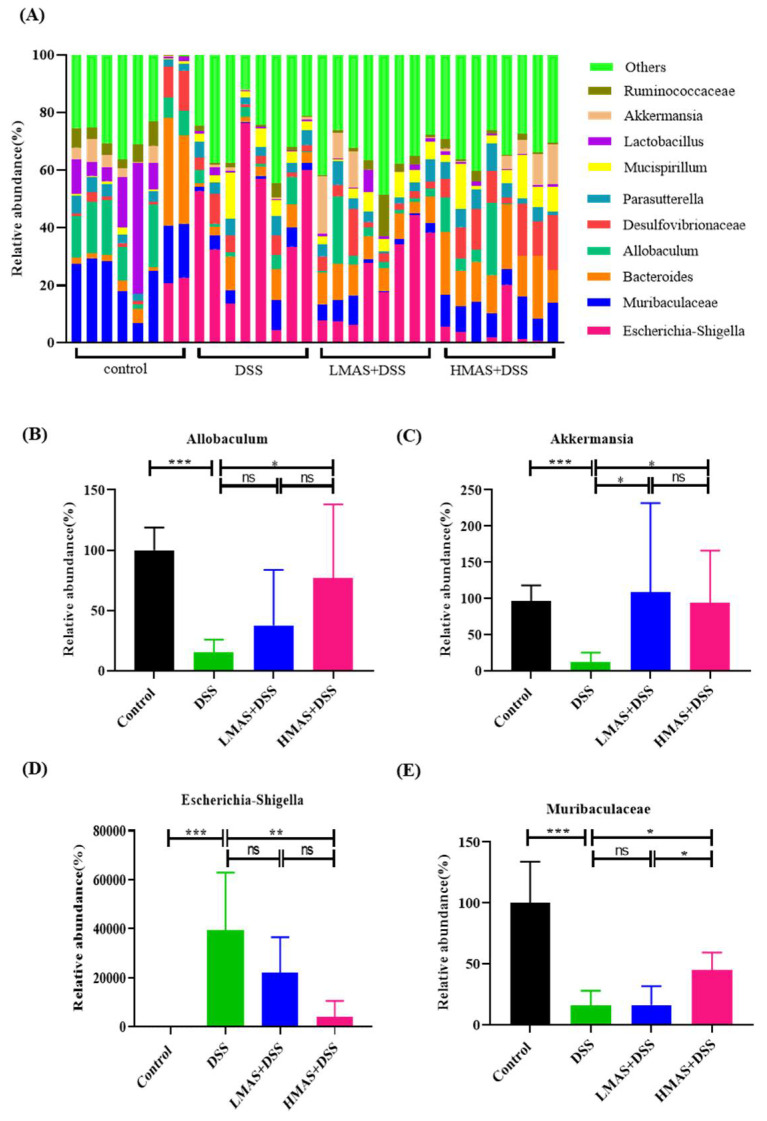
MAS regulates the gut microbiota composition at the genus level. (**A**) Histogram of the relative abundance of genera among the different groups. (**B**–**E**) MAS regulated *Allobaculum*, *Akkermansia*, *Escherichia-Shigella*, and *Muribaculaceae*. Here, ns = not significant. Data are shown as the mean ± SD. * *p* < 0.05, ** *p* < 0.01, *** *p* < 0.001, in comparison with the DSS group (n = 8).

We also analyzed the abundances of intestinal microbiota at the genus level. As shown in Figure 6, the relative abundances of *Muribaculaceae* (*p* < 0.001), *Allobaculum* (*p* < 0.05), and *Akkermansia* (*p* < 0.001) decreased greatly and *Escherichia-shigella* increased significantly in the DSS group compared to those in the control group. However, MAS treatment can significantly reverse these changes (*p* < 0.05). In brief, these obvious changes in gut microbiota at the genus level under the intervention of MAS not only confirmed the regulatory effect of MAS on the gut microbiota but also implied that these genera might be important regulatory bacteria in the process of MAS exerting its effects.

### 3.7. Linear Discriminant Analysis (LDA) Integrated with Effect Size (LEfSe)

**Figure 7:**
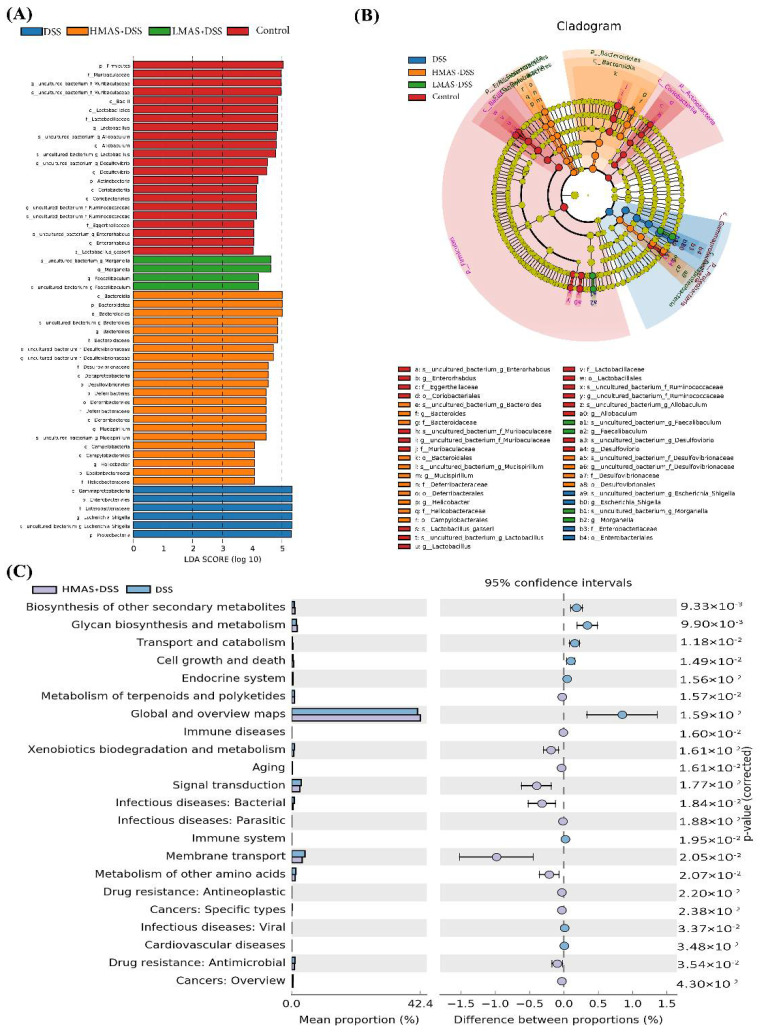
MAS regulates the bacterial metabolic pathway. (**A**) LDA analysis. (**B**) LEfSe analysis. (**C**) Analysis of differences in KEGG metabolic pathways between groups.

To discriminate the intestinal bacterial communities among these four groups, LEfSe analysis was then performed to figure out the specific microbes associated with MAS treatment (Figure 7A,B). *Firmicutes* and *Muribaculaceae* were abundant in the control group. The opportunistic pathogenic bacteria in Staphylococcus genus such as *Gammaproteobacteria*, *Enterobacteriales*, *Enterobacteriaceae* and *Eschericha_Shigella* were overrepresented in the DSS group, while the higher abundance of potentially beneficial microbes such as *Bacteroides*, *Bacteroidetes* and *Bacteroidales* as the top three taxa were observed in the high dose of MAS treatment. In conclusion, this result indicates that colitis can significantly disturb the structure and composition of the intestinal microbiota, which were inhibited, and the balance was restored after the MAS treatment.

We then used the PICRUSt analysis and the KEGG orthology to investigate the effect of MAS on potential metabolic pathways in the gut microbiota of colitis mice [32]. As shown in Figure 7C, we found that the metabolism of the intestinal microbiota of the DSS group was significantly abnormal, for example, the ability of biosynthesis of other secondary metabolites and glycan biosynthesis and metabolism pathways of bacteria were decreased, and the ability of signal transduction and membrane transport of bacteria were increased in the DSS group. Interestingly, MAS treatment can significantly correct these abnormal changes. In a word, MAS could improve gut microbiota dysfunction in DSS-induced colitis.

## 4. Discussion

It is well recognized that UC is associated with dysbiosis in the intestinal microbiota of patients and mice [33,34]. The modulation of intestinal flora disorders has become a promising strategy for the treatment of intestinal inflammation. Dietary intervention in the treatment of UC has been increasingly appreciated over recent years [35,36,37]. Recent evidence suggests that mulberry intake is supposed to be negatively correlated with both inflammation activity and oxidative stress [14]. However, the protective effects of MAS against UC by regulating gut microbiota have not yet been fully explored and understood. Firstly, we identified two main components in this prescription by the HPLC method, mainly including Cyanidin-3-O-glucoside and Cyanidin-3-O-rutinoside, which have been proven to possess high bioavailability and antioxidant activity [38,39]. However, it is unknown whether MAS may exert anti-inflammation and intestinal microbiota regulation properties in colitis. We conducted a DSS-induced colitis model to study UC and found that MAS was enough to ameliorate colitis by reducing the body weight loss, disease activity index (DAI) scores, colon length shortening and intestinal permeability. Moreover, other mechanisms of MAS in UC have been proposed, including maintaining the integrity of the gut barrier and reshaping the gut microbiota composition for MAS treatment.

Cytokines are involved in the development of UC [40]. Pro-inflammatory cytokines, including Il-6, IFN-α, and IL-1β, are the main characteristics of colitis. Our results showed that pro-inflammatory cytokine levels were elevated in the DSS group. However, MAS treatment significantly inhibited the secretion of pro-inflammatory cytokines. In addition, previous studies have reported that the anti-inflammatory cytokine IL-10 ameliorates intestinal damage in UC [41]. In accordance with these studies, our results indicated that IL-10 keeps a higher level in the HMAS-DSS group (Figure 2F). These results indicated that MAS may have important effects on gut anti-inflammatory modulators in mice with DSS-induced colitis.

Oxidative stress is the underlying mechanism of IBD pathophysiology [42]. The endogenous self-antioxidant defenses are disrupted with the production of excessive ROS, leading to mucosal rupture and ulceration infiltrated by massive inflammatory cells in the colon tissues [43]. High concentrations of ROS attack and inactivate endogenous antioxidant factors, such as GSH, SOD, and CAT, thus preventing their effective neutralization of ROS. In the present study, the GSH, SOD, and CAT activities of colon tissue were significantly reduced in DSS-treated mice (Figure 2G), which is consistent with previous reports [44,45]. Notably, MAS administration significantly inhibited the elevation of colonic MDA activity and restored colonic GSH, SOD, and CAT activities. Taken together, these findings suggest that MAS may have the ability to inhibit oxidative stress via exerting antioxidant capacity.

Epithelial surfaces in the gut are covered by a layer of mucus, as a physical barrier, which prevents bacteria from accessing the mucosal surface [46]. Muc2 is exclusively secreted by intestinal goblet cells and is the main component of intestine mucus [47]. Muc2-deficient mice spontaneously contribute to the development of colitis and colorectal cancer, and at the same time, the microbial composition is different from that in wild-type mice [48]. The immunofluorescence analysis in this study revealed MAS stimulated Muc2 expression in intestinal goblet cells after DSS treatment. The intestinal epithelial TJ barrier and intestinal permeability are crucial for maintaining intestinal homeostasis [49]. Intestinal epithelial cells, mucin layers, and TJ proteins contribute to intestinal permeability [50]. In addition, previous studies have found that alterations in TJ proteins in colonic epithelial cells can significantly exacerbate intestinal colitis [51]. Our results have shown a decrease in TJ protein and an impairment of intestinal epithelial integrity in DSS-induced colitis mice. However, MAS may relieve the changes in tissue structure and intestinal integrity by upregulating TJ protein expression.

Many studies have shown that dysbiosis of the intestinal microbiota plays a fundamental role in the progression of UC. The alpha-diversity and structure of the intestinal microbiota in mice with DSS-induced UC were disrupted [40]. In line with these studies, we also found reduced bacterial abundance and richness in the DSS model group compared to the control group, and high doses of MAS treatment maintained alpha-diversity (Figure 5). In addition, beta-diversity comparisons reflect the taxonomic similarity between different samples [52]. The control and HMAS+DSS groups clustered more closely together, while the DSS group deviated more from these groups. The above results indicate that MAS treatment maintained the intestinal microbiota composition and that there was significant variability in diversity from the DSS treatment.

The main core of the gut microbiota is composed of *Bacteroidetes* and *Firmicutes* [53]. The intestinal microorganisms of colitis showed a reduction in the *Firmicutes*/*Bacteroidetes* ratio, leading to reduced biodiversity and dysbiosis in colitis patients [54]. In this study, the change of *Firmicutes*/*Bacteroidetes* ratio was inhibited when MAS was continuously administrated (Figure 5). At the same time, the *Proteobacteria* was significantly increased in DSS-induced colitis. MAS treatment inhibited the relative abundance of the *Proteobacteria*, which is thought to be associated with the regulation of epithelial dysfunction [55].

Previous studies have shown that the development of colitis leads to an increase in *Escherichia-Shigella,* causing gut microbiota disturbance [40]. We observed a significant increase in the abundances of *Escherichia-Shigella* in DSS-induced mice at the genus level, which was controlled to similar levels observed in the control group under MAS intervention. It is worth noting that our data show that colitis reduced the abundance of *Akkermansia*, while MAS treatment recovered its abundance. *Akkermansia* is a type of probiotic, representative of the probiotic family and phylum [56]. *Akkermansia* is a common gut microbiota, whose main function is to degrade host mucin into various products (e.g., short-chain fatty acids) to maintain intestinal barrier function and regulate immune responses [57]. At the same time, the consumption of many proanthocyanidin-rich substances such as blueberry polyphenol extracts and grape polyphenols can also increase relative the proportion of intestinal *Akkermansia* [58]. Therefore, from this perspective, major functions of MAS in the gut appear to be its role as a key intermediate in the maintenance of epithelial integrity and preservation of barrier function through increasing the *Akkermansia* abundance.

*Allobaculum* also is the most abundant in the intestinal microbiota, accounting for 40.42% of the total number of bacteria at the genus level [59], which was reduced after DSS treatment. In addition, *Allobaculum* plays a protective role in shaping adult metabolism and anti-inflammation [60,61]. Thus, *Allobaculum* may be an important genus negatively associated with UC, and MAS treatment alleviates UC by increasing *Allobaculum* abundance to reduce the risk of UC in mice.

Taken together, this study demonstrated that MAS treatment inhibited DSS-induced clinical symptoms and colonic damage, reduced intestinal inflammation and oxidative stress, restored intestinal barrier integrity, and maintained immune homeostasis. Meanwhile, MAS enhanced barrier function by regulating the structure of intestinal microbiota by reducing the level of potentially harmful bacteria (*Escherichia-Shigella*) and enriching the relative abundance of potentially beneficial bacteria (*Allobaculum*, *Akkermansia* and *Muribaculaceae*). The functional COG and KEGG databases for predicting intestinal bacteria have also been changed depending on the composition of the intestinal microbiota. These results suggest that MAS exerts an anti-UC effect by improving intestinal flora disorders, suggesting that MAS may be used as a dietary component in the prevention and treatment of colitis.

## 5. Conclusions

The results suggest that the benefits of mulberry anthocyanin extract on colitis may be mainly via regulating the changes in the structure of gut microbiota, revising intestinal inflammation, oxidative stress, and the integrity of the intestinal barrier. All results indicate that mulberry anthocyanins positively contribute to reducing the inflammatory response. Overall, the study suggests that consumption of mulberry anthocyanins may be functional ingredients to prevent or lessen the symptoms of IBD.

## Data Availability

The data are contained within the article.
